# A Memorial of Midwifery, Etc.

**Published:** 1871-10

**Authors:** 


### BOOK NOTICES.

A Memorial of Midwifery, including the Signs and Symptoms of Pregnancy, Obstetric Operations, Diseases of the Puerperal State, etc., etc. By Alfred Meadows, M. D. London. First American, from the second London edition. Published by Lindsay & Blakiston, Philadelphia. For sale by S. C. Griggs, Chicago.

This is a small octavo volume of 473 pages. In the first part the physiology of conception and gestation, with the development of the ovum from the time it leaves the ovisæ to its full maturity, together with the coincident changes occurring in the uterus, are considered. The second part embraces the whole subject of pregnancy: its signs and symptoms, its duration, and the various deviations from what may be called normal pregnancy, including the various forms of extra-uterine gestation and of displacements of the gravid uterus.

The third part treats of natural parturition, the classification of labors, and the phenomena and management of natural labors. The fourth part brings under review the various obstetric operations which are necessitated by the different emergencies described and illustrated in Parts V. and VI, under the heads of unnatural and complex labor. Lastly, some of the principal diseases of the puerperal state are described in Part VII.

This systematic arrangement of subjects, and the concise, practical style in which it is written, make the work especially valuable as a students' manual, while a very full table of contents and index render it easily accessible as a work of reference.

